# Association between depression and macrovascular disease: a mini review

**DOI:** 10.3389/fpsyt.2023.1215173

**Published:** 2023-06-29

**Authors:** Shuwu Zhao, Liping Zhu, Jinfeng Yang

**Affiliations:** ^1^Department of Anesthesiology, Hunan Cancer Hospital, The Affiliated Cancer Hospital of Xiangya School of Medicine, Central South University, Changsha, China; ^2^Department of Rehabilitation Medicine, The 3rd Xiangya Hospital, Central South University, Changsha, China

**Keywords:** depression, macrovascular disease, cerebrovascular disease, coronary artery disease, platelet dysfunction, proinflammatory cytokines

## Abstract

Depression and macrovascular diseases are globally recognized as significant disorders that pose a substantial socioeconomic burden because of their associated disability and mortality. In addition, comorbidities between depression and macrovascular diseases have been widely reported in clinical settings. Patients afflicted with coronary artery disease, cerebrovascular disease or peripheral artery disease exhibit an elevated propensity for depressive symptoms. These symptoms, in turn, augment the risk of macrovascular diseases, thereby reflecting a bidirectional relationship. This review examines the physiological and pathological mechanisms behind comorbidity while also examining the intricate connection between depression and macrovascular diseases. The present mechanisms are significantly impacted by atypical activity in the hypothalamic–pituitary–adrenal axis. Elevated levels of cortisol and other hormones may disrupt normal endothelial cell function, resulting in vascular narrowing. At the same time, proinflammatory cytokines like interleukin-1 and C-reactive protein have been shown to disrupt the normal function of neurons and microglia by affecting blood–brain barrier permeability in the brain, exacerbating depressive symptoms. In addition, platelet hyperactivation or aggregation, endothelial dysfunction, and autonomic nervous system dysfunction are important comorbidity mechanisms. Collectively, these mechanisms provide a plausible physiological basis for the interplay between these two diseases. Interdisciplinary collaboration is crucial for future research aiming to reveal the pathogenesis of comorbidity and develop customised prevention and treatment strategies.

## 1. Introduction

Because of the rapid progression of society and quick pace of modern life, people of all ages experience enormous amounts of mental stress. Depression, which is a highly prevalent psychiatric disorder (1), is characterized by sustained feelings of sadness, gloom or emptiness; sleep disturbances; increased guilt; feelings of self-reproach, helplessness and anxiety; decreased social and personal efficacy; and weight changes or altered appetites, all of which significantly impact daily life (2, 3). Research shows that 28.48% of college students have clinical depression (4). Similarly, depression also exists in 17–53% of outpatients (5) and in 35.1% of the elderly (6). It is particularly noteworthy that 6% of the population suffers from major depression (7). Depression is characterized by its persistent and recurring nature, which poses a severe hazard to human well-being. Depression is a leading aetiology for disability, and its economic impact on society is considerable (8–10). As time goes on, the economic cost of depression is expected to continue to rise and is projected to double by 2030 (3). Although certain factors, such as neurotransmitter imbalance, immune inflammatory response, and genetics, contribute to depression (11, 12), the specific pathological and physiological mechanisms underlying this disorder remain unclear.

MVD refers mainly to diseases of the large blood vessels, including the coronary arteries, aorta and larger arteries of the brain and limbs (13). MVD is intricately linked to a range of factors, including oxidative stress, inflammatory reactions, genetic predisposition, platelet dysfunction and endothelial impairment (14–18). Elevated cholesterol levels and hyperlipidaemia have been observed to potentially induce endothelial dysfunction through the oxidized low-density lipoprotein, thereby precipitating an imbalance in arterial constriction and dilation before ultimately inciting the development of atherosclerosis (19). Platelet dysfunction may facilitate the advancement of atherosclerosis via the secretion of chemokines and recruitment of leukocytes (receptor–ligand interactions) (20, 21). It is noteworthy that certain genetic factors may also play a role in the aetiology of MVD. To date, a number of genetic loci, including chromosome 9 p21 (chr9p21), alpha 1-3-N-acetylgalactosaminyltransferase and alpha 1-3-galactosyltransferase (ABO), have been identified as being linked to MVD (18). Recent studies have proposed an association between gut microbial dysbiosis (e.g., its metabolites trimethylamine N-oxide, short-chain fatty acids and taurine) and cerebrovascular disease (22). According to clinical practice, the common pathogenic factors of MVD are not single, but instead, they involve a combination of factors (23).

Several investigations have demonstrated a significant correlation between depression and certain MVD, which often manifest as comorbidities (24–28). For instance, research findings indicate that the prevalence rate of depressed patients with peripheral artery disease (PAD) lies between 16 and 35%, and for people with both PAD and depression, the risk of death increases by 24% (29). Similarly, roughly one-third of coronary artery disease (CAD) patients experience depression (30), increasing their cardiovascular mortality risk by 31% (31). In addition, poststroke depression affects 27% of stroke patients and correlates with a 25% mortality rate (32, 33). However, research on the relationship between aortic diseases and depression remains relatively limited. It should be noted that depression and MVD are not unidirectional but often influence each other, showing a bidirectional relationship between them that increase each other’s risks. For instance, the emergence of depression is attributed to vascular risk factors, while the occurrence of MVD is heightened by 30% in individuals experiencing concurrent depression (33–36). Despite significant strides made in unraveling the pathogenesis of depression and MVDs, our understanding of their interplay and underlying mechanisms remains in its infancy. Accordingly, the present review seeks to comprehensively elucidate the potential mechanisms that underscore the interplay between MVD and depression, with the intent of furnishing theoretical underpinnings for optimizing clinical interventions.

## 2. Risk factors

**Figure 1:**
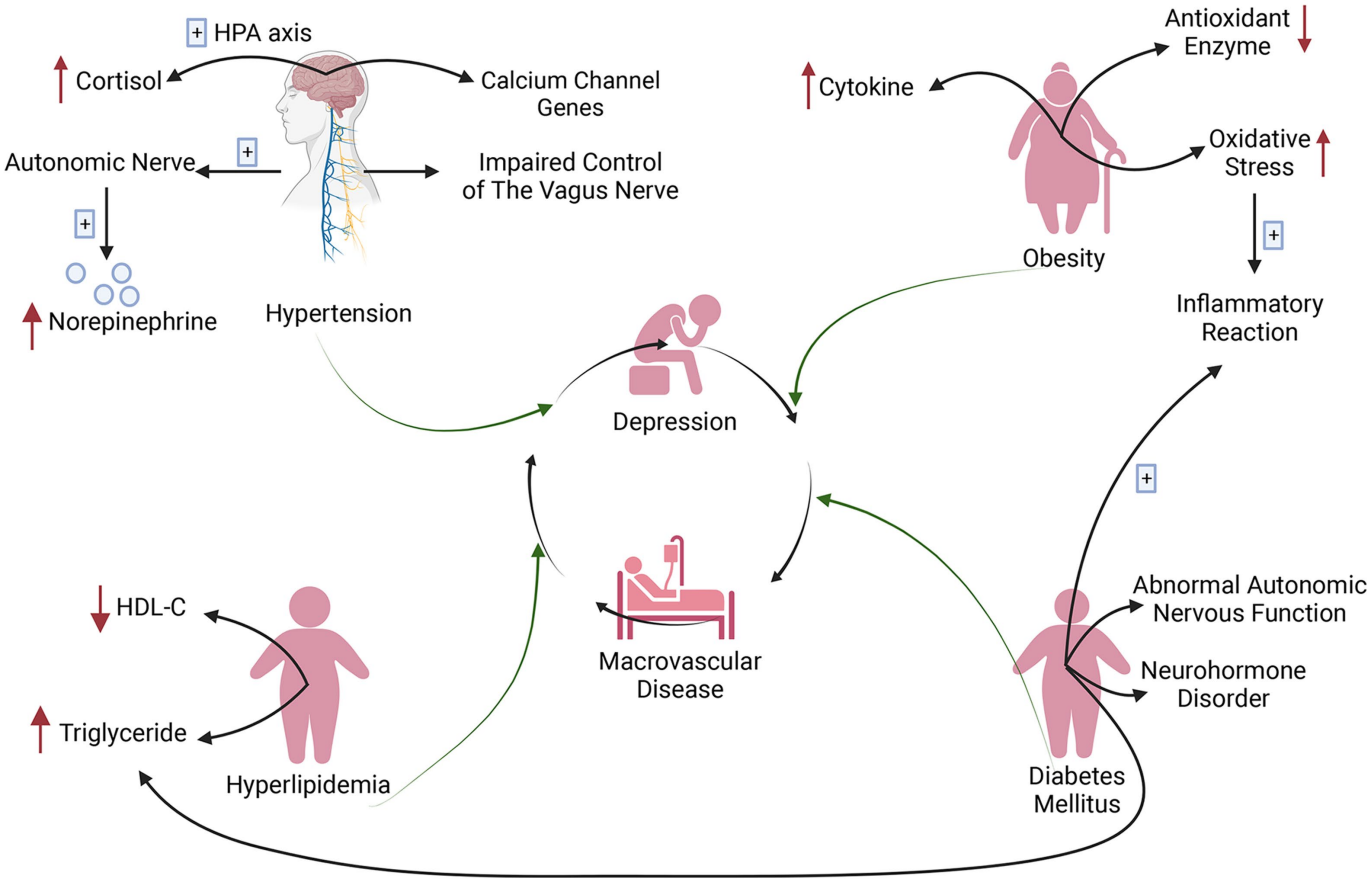
Risk factors for comorbidity of depression and macrovascular disease (created with BioRender.com). Calcium channel genes, autonomic nervous system abnormalities (increased norepinephrine) and increased cortisol (hypothalamic–pituitary–adrenal axis abnormalities) are all associated with macrovascular disease and depression in hypertension, and impaired vagal control is an important factor mediating both diseases. Obese patients amplify oxidative stress to promote an inflammatory response that mediates the development of diabetes mellitus. Neurohormonal dysregulation and abnormal autonomous nerve function in diabetic patients influence the development of depression and macrovascular disease. Diabetic metabolic dysregulation, that is, increased triglycerides and decreased hypothalamic–pituitary–adrenal cholesterol, causes hyperlipidaemia, which further contributes to depression and macrovascular disease because of atherosclerosis. HDL-C, high-density lipoprotein cholesterol; HPA, hypothalamic–pituitary–adrenal.

Comorbidity refers to the aggregation of multiple diseases within specific populations because of their biological, social and environmental interactions, which can result in an overall increase in the disease burden (30). Depression and MVD share similar psychosocial and physical factors in specific populations and manifest in the form of comorbidity (Figure 1). In the complex interaction between depression and MVD, hypertension, hyperlipidaemia, diabetes mellitus (DM), and obesity have been identified as significant mediators (36–39).

Adipose tissue secretes cytokines, which are carried to the brain by the blood and affect the neurotransmitter system, ultimately mediating the occurrence of depression (37, 40). Additionally, obesity can significantly exacerbate vascular risk factors such as high blood pressure and lipid abnormalities, thereby resulting in negative effects on vascular structure and function (41). Research has demonstrated that obesity contributes to the development of stroke by amplifying oxidative stress and lowering antioxidant enzyme levels, while oxidative stress induces depressive symptoms by promoting oxidation and inflammation through reactive oxygen species (42).

However, certain investigations have not discovered a correlation between depressive symptoms and clinical factors such as hypertension and hyperlipidaemia (43). A plethora of other research has indicated that hypertension, hyperlipidaemia and DM are not only the traditional risk factors of MVD, but they are also intricately linked to the emergence and advancement of depression. Of type 2 diabetes mellitus (T2DM) patients, 10.6% have comorbid major depression, increasing cardiovascular morbidity and mortality (44). Major depression affects 27% of hypertension patients, heightening their susceptibility to stroke events and heart failure. This link may be attributed to compromised blood pressure regulation (45, 46). Patients suffering from depression and pre-existing CAD may have diminished cardiac regulation, which can result in hypertension (46).

Impaired control of the vagus nerve may mediate the connection between depression and hypertension (47). Studies have shown that calcium channel genes, autonomic nervous system abnormalities (increased norepinephrine) and increased cortisol are all associated with MVD and depression in hypertensive patients (45, 46, 48). Depressed patients frequently exhibit low levels of high-density lipoprotein cholesterol (HDL-C) and elevated triglyceride levels, both of which promote the growth of atherosclerosis, which is a key driver of vascular disease (49). In addition, autonomic nervous dysfunction and neurohormone imbalance, inflammatory reaction and hippocampal structural alterations are all significant factors that contribute to the complex and bidirectional association between DM and depression (40, 50). Elevated triglyceride levels resulting from metabolic dysregulation in DM can cause vascular dysfunction, increase the risk of atherosclerosis and predispose individuals to coronary heart disease (51). Moreover, patients suffering from metabolic syndrome are at an elevated risk of developing depression (52).

## 3. Pathophysiological mechanism

**Figure 2:**
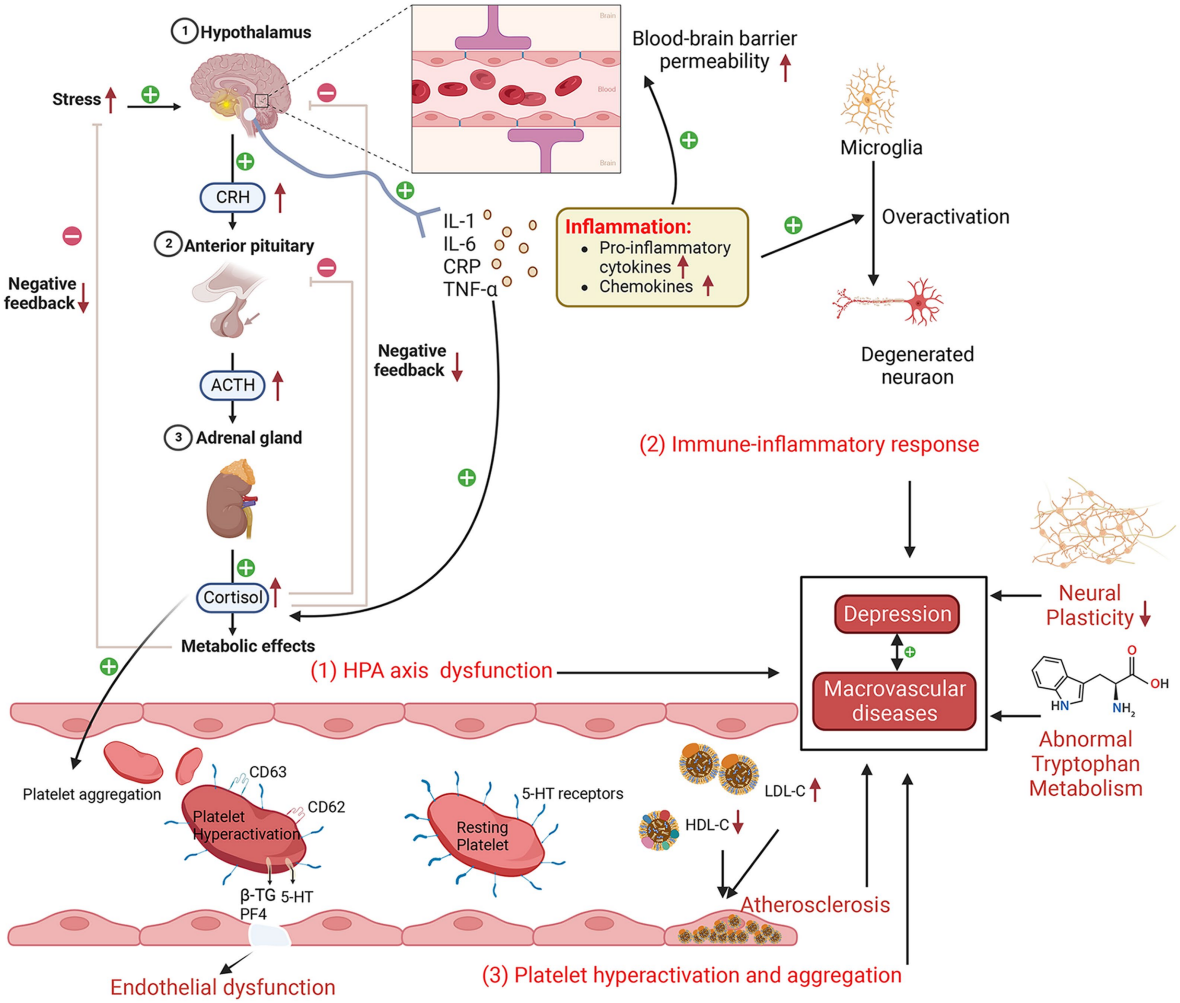
Schematic diagram of the possible comorbidities of depression and macrovascular disease (created with BioRender.com). Depressive episodes are characterized by increased hypothalamic–pituitary–adrenal axis activity, elevated cortisol levels, an enhanced response to corticotropin-releasing hormone, increased adrenocorticotropic hormone, and decreased cortisol feedback inhibition. Subsequently, increased secretion of hormones, such as cortisol, can damage vascular endothelial cells, leading to endothelial dysfunction and an increased inflammatory response. Proinflammatory cytokines affect cerebral blood–brain barrier permeability, disrupt microglia and neuronal function, and promote cortisol secretion, which, in turn, affects hypothalamic–pituitary–adrenal axis function. Platelet hyperactivation and aggregation lead to increased endothelial adhesion, resulting in endothelial dysfunction. In addition, abnormal tryptophan metabolism and reduced neuroplasticity play a role in depression and macrovascular disease. HPA, hypothalamic–pituitary–adrenal; LDL-C, low-density lipoprotein cholesterol; IL-6, interleukin-6; CRP, C-reactive protein; CRH, corticotropin-releasing hormone; IL-1, interleukin-1; TNF-α, tumor necrosis factor-α; 5-HT, serotonin; HDL-C, high-density lipoprotein cholesterol; PF4, platelet factor 4; β-TG, β-thromboglobulin; ACTH, adrenocorticotropic hormone.

There has been an upsurge in scholarly studies exploring the bidirectional interplay and fundamental mechanisms linking depression with various vascular diseases including CAD, cerebrovascular disease (CVD) and PAD. Currently, research on their comorbidity mechanisms primarily centers on three aspects (Figure 2): neuroendocrine factors, immune inflammatory responses and platelet dysfunction (53). The mechanisms in question not only precipitate and exacerbate depression but also contribute to the development of numerous complications associated with these vascular diseases.

### 3.1. Neuroendocrine factors

One of the current top research priorities in neuroendocrinology is the aberrant function of the hypothalamic–pituitary–adrenal (HPA) axis, which is a pathogenic mechanism implicated in depression (54), as evidenced by the fact that taking antidepressants can restore the abnormal HPA axis (55). Some scholars believe that the aberrant functioning of the HPA axis may represent a contributory cause for the comorbidity of depression and CAD/CVD (56, 57). Some studies have found a significant upregulation of HPA axis functioning in individuals with depression, which consequently results in overstimulation of the autonomic nervous system. This is clinically manifested by increased basal cortisol levels, an enhanced response to corticotropin-releasing hormone (CRH), increased adrenal cortical hormone (ACTH) secretion, and decreased cortisol feedback inhibition (58–61). In addition, increased secretion of hormones such as cortisol can further damage vascular endothelial cells, leading to abnormal endothelial cell function, increased injury and inflammatory reactions, ultimately causing vascular injury, constriction and narrowing, resulting in the occurrence of MVD (62–65).

### 3.2. Immune inflammatory reaction

Some studies have demonstrated an elevation in inflammatory markers, including interleukin-1 (IL-1), interleukin-6 (IL-6), C-reactive protein (CRP), and tumor necrosis factor-α (TNF-α), among patients afflicted with depression (40, 66–68). These findings provide compelling evidence for the involvement of immune inflammatory reactions in the pathogenesis of depression (69). Moreover, some studies have found that anti-inflammatory treatment can improve depression-like behavior in mice, thereby reinforcing the notion of inflammation as a critical contributor to developing depression (70). The release of proinflammatory cytokines has been found to disturb blood–brain barrier permeability and initiate inflammatory cascades that amplify inflammatory signals. Additionally, these cytokines have been observed to result in functional abnormalities in microglia and neurons, contributing to the exacerbation of depressive symptoms (66, 69, 71–73). Moreover, cytokine activation promotes the upregulation of adhesion molecules on endothelial cells, which then drives the infiltration of monocytes and lymphocytes into the vessel wall. This, in turn, triggers a localized inflammatory response within the vascular lining, ultimately fostering the progression of atherosclerosis and emergence of MVD, such as CVD and CAD (74, 75). Additionally, some cytokines can promote the release of ACTH, CRH and cortisol, thereby affecting the regulatory function of the HPA axis (76, 77). It is worth noting that TNF-α has been identified as a biomarker of inflammatory reactions after brain injury (78). It has also been reported that TNF-α can either indirectly or directly lead to apoptosis and then to vascular calcification and injury (14, 79, 80). IL-6 has been found to be critical in the pathophysiology of brain injury because it can enhance glial cell activation and stimulate endothelial cells to produce adhesion molecules (81). The aforementioned studies indicate that immune inflammatory responses exert a crucial effect on the pathogenesis of depression and MVD. Despite the substantial advancements made in comprehending the link between inflammation and these two disorders, further investigations are warranted to elucidate the precise underlying mechanisms. The aforementioned study outcomes will significantly contribute to our comprehension of the interrelation between these diseases and assist in advancing more potent strategies for their prevention and treatment.

### 3.3. Platelet dysfunction

Some studies have shown abnormal platelet aggregation and coagulation functions in depression and MVD (16, 82–84). A study based on flow cytometry shows that diabetic patients presenting with concomitant signs of depression exhibited platelet hyperactivation (indicated by platelet activation markers CD 62 and CD 63) compared with those without depression symptoms (85). Moreover, studies have demonstrated that patients affected by depression display increased platelet activity, as assessed via the mean platelet volume, when compared with control groups without depression symptoms (86, 87). This phenomenon may be linked to elevated secretion levels of cortisol and catecholamines in the autonomic nervous system (88, 89). Platelet dysfunction can lead to a series of problems, including increased responsiveness to physiological stress, which, in turn, causes continuous enhancement of platelet activation and aggregation (90). This then causes a rise in platelet adhesion towards the vascular endothelium (91), promoting thrombosis and vascular stenosis, ultimately triggering MVD. During platelet activation, vascular endothelial cells discharge an array of chemokines and cytokines from α-granules, including platelet factor 4 (PF4), β-thromboglobulin (β-TG), and serotonin (5-HT), which enhance inflammatory reaction and thrombosis (83, 92). The pivotal contribution of 5-HT to the onset of depression has been well established (89). As a prominent neurotransmitter, 5-HT is capable of binding with 5-HT receptors located on the platelet surface, augmenting their aggregation process, which requires the participation of the 5-HT transporter protein (83, 93, 94). Therefore, the potential utility of serotonin as a promising pharmacological target for treating depression has been established, notably through the use of selective serotonin reuptake inhibitors (SSRIs) (94–96), which have been confirmed to effectively curtail platelet activation, consequently alleviating the risk of myocardial infarction (97).

### 3.4. Other mechanisms

The endothelium, a monolayer of cells lining the inner surface of blood vessels, is essential for maintaining vascular function by regulating vessel expansion and contraction, inhibiting platelet aggregation, and preventing leukocyte adhesion (98). Endothelial dysfunction occurs when the physiological function of endothelial cells becomes impaired, which leads to vasoconstriction, elevated cytokine levels and platelet and leukocyte aggregation (99). It is noteworthy that endothelial dysfunction has been regarded as a characteristic marker of depression and atherosclerosis (100, 101), indicating that endothelial dysfunction is one of the pathophysiological factors of depression and MVD (99, 102, 103). Specifically, the flow-mediated dilatation value (which is used to evaluate endothelial function) of depressed patients is lower than that of nondepressed patients (104). This has also being observed in PAD (105).

For the comorbidity mechanism of depression and MVD, some scholars have put forward the vascular depression hypothesis (106). According to this hypothesis, patients affected by MVD frequently manifest depressive features attributed to cerebral white matter lesions, which augment low-density lipoprotein cholesterol levels and decrease high-density lipoprotein cholesterol levels, thereby exacerbating the severity of MVD (107, 108). In addition, other possible mechanisms contribute to the comorbidity of depression and MVD, including decreased neural plasticity (109), autonomic dysfunction (decreased heart rate variability) (110) and abnormal tryptophan metabolism (111).

## 4. Relationship between specific macrovascular disease and depression

### 4.1. Coronary artery disease

The marked correlation between CAD and depression has garnered widespread attention. Depression has been determined to be a standalone danger element for CAD (112). According to the findings of a cohort study, individuals exhibiting depression may be at an increased risk of developing ischaemic heart disease. Specifically, those displaying mild symptoms have shown a 1.5-fold higher likelihood than their nondepression counterparts, and those with severe symptoms had an even greater elevated risk of 1.6-fold (110, 113). For patients already burdened with ischaemic heart disease, depressive symptoms lead to an elevated risk of 11% for vascular events and a 23% rise in all-cause mortality (114, 115). Ricardo de Miranda Azevedo et al. (116) suggest that depressive symptoms have demonstrable ramifications for the prognostication of myocardial infarction patients, with notable implications that are contingent on both gender and age. More critically, a retrospective cohort study conducted in China encompassing nationwide heart failure patients indicated that among hospitalized heart failure patients, those with depression were more likely to be readmitted within 30 days (117).

### 4.2. Cerebrovascular disease

In addition to CAD, an unequivocal correlation between depression and CVD has been repeatedly demonstrated (118). According to a meta-analysis, stroke victims who have poststroke depression have a higher risk of having their strokes reoccur (119), with an incidence rate between 18 and 33% (120, 121). Patients with depression exhibited a 3.18% amplified risk of experiencing transient ischaemic attack (TIA) relative to patients without depression. Furthermore, for patients who have previously undergone TIA, there is a 6.88% likelihood of developing depressive symptoms (122). Patients with CVD who are depressed have a worse prognosis and quality of life. Several investigations have demonstrated that patients who suffer from stroke manifest depressive symptoms, exhibit a comparatively inferior quality of life, have heightened rates of disability, are more likely to attempt suicide, and have elevated rates of mortality relative to those stroke patients absent of depression (39, 123–126), with an all-cause mortality risk ratio of 1.59 (125).

### 4.3. Peripheral arterial disease

PAD and depression are common comorbidities. Depressive symptoms have been shown to afflict 16–35% of PAD patients, which increases the risk of mortality by 24% (29). According to a meta-analysis covering 20 studies, the prevalence of depression in patients with PAD was estimated to be 13%. This comorbidity has been found to augment the risk of adverse limb consequences, notably intermittent claudication, by 20%; at the same time, these patients are accompanied by more complications such as chest pain and palpitations (127, 128). In addition, PAD patients afflicted with depression are characterized by heightened risks of amputation and mortality, decreased walking function and a lower quality of life (29, 128–130). A nationwide observational study covering 155,647 retired military personnel with PAD revealed that patients suffering from coexisting depression heightened the risk of amputation by 13% and the risk of mortality by 17% in contrast to their counterparts without any comorbid depression (131). These findings indicate that the early identification, evaluation and management of depression in PAD patients are of great significance.

## 5. Conclusion and prospect

Multiple studies have established a strong link between depression and MVD. Depression affects the prognosis of MVD, while MVD further exacerbates depressive symptoms, indicating a bidirectional relationship between them. This review highlights the complex and diverse pathophysiological mechanisms underlying the comorbidity of depression and MVD, including neuroendocrine factors (HPA axis dysfunction), immune inflammatory responses (proinflammatory cytokines) and platelet dysfunction (hyperactivation and aggregation). In addition, the interaction between them is also influenced by pathological elements including endothelium dysfunction and autonomic nervous system dysfunction. The review has also elucidated the relationships between depression and CAD, CVD, and PAD. These studies on correlations provide a theoretical basis for interdisciplinary collaboration to achieve a better therapeutic effect.

Despite the clinical recognition of comorbidity between depression and MVD, additional studies are required to gain a comprehensive understanding of the underlying pathophysiological mechanisms. Future studies should aim to dive deeper into the mechanisms of interaction between these diseases, including possible pathological, physiological and molecular mechanisms.

## Author contributions

SZ and LZ conceived and wrote the article. JY revised and reviewed the article. All authors contributed to the article and approved the submitted version.

## Funding

This work was supported by grants from the Science and Technology Innovation Program of Hunan Province (No. 2021SK4014) and the Hunan Cancer Hospital Climb Plan.

## Conflict of interest

The authors declare that the research was conducted in the absence of any commercial or financial relationships that could be construed as a potential conflict of interest.

## Publisher’s note

All claims expressed in this article are solely those of the authors and do not necessarily represent those of their affiliated organizations, or those of the publisher, the editors and the reviewers. Any product that may be evaluated in this article, or claim that may be made by its manufacturer, is not guaranteed or endorsed by the publisher.
